# C-Terminal Di-leucine Motif of Dopamine D_1_ Receptor Plays an Important Role in Its Plasma Membrane Trafficking

**DOI:** 10.1371/journal.pone.0029204

**Published:** 2011-12-19

**Authors:** Yan Guo, Pedro A. Jose

**Affiliations:** Center for Molecular Physiology Research, Children's National Medical Center, Washington, D. C., United States of America; University of Houston, United States of America

## Abstract

The dopamine D_1_ receptor (D_1_R), a G protein-coupled receptor, plays a critical role in regulating blood pressure through its actions on renal hemodynamics and epithelial ion transport, which are highly linked to its intracellular trafficking. In this study, we generated a series of C-terminal mutants of D_1_R that were tagged with or without enhanced yellow fluorescent protein, and analyzed the consequences of these mutants on the plasma membrane trafficking of D_1_R and cyclic AMP response to D_1_R stimulation. D_1_R with mutations within the endocytic recycling signal (amino acid residues 360–382) continued to be functional, albeit decreased relative to wild-type D_1_R. Mutation of the palmitoylation site (347C>S) of D_1_R did not impair its trafficking to the plasma membrane, but abolished its ability to increase cyclic AMP accumulation. In contrast, replacement of di-leucines (344–345L>A) by alanines resulted in the retention of D_1_R in the early endosome, decreased its glycosylation, and prevented its targeting to the plasma membrane. Our studies suggest that di-L motif at the C-terminus of D_1_R is critical for the glycosylation and cell surface targeting of D_1_R.

## Introduction

Dopamine, produced in the kidney, known to play an important role in regulating renal sodium excretion [1], produces its biological effects through five genetically distinct dopamine receptors in mammals [2]. It has been reported that defective dopamine receptor function, especially the dopamine D_1_ receptor (D_1_R), in the kidney is found in humans with essential hypertension [3]. Deletion of any of the dopamine receptor genes, including the D_1_R, in mice produces hypertension, the pathogenesis of which is specific to the particular dopamine receptor subtype [4], [5]. Dopamine receptors belong to a large family of G protein-coupled receptors (GPCRs) that sense molecules outside the cell and activate inside signal transduction pathways and, ultimately, cellular responses. There are two principal signal transduction pathways involving GPCRs: the cyclic AMP (cAMP) pathway and the phosphatidylinositol pathway [6]. Based on their ability to stimulate or inhibit adenylyl cyclase, dopamine receptors are classified into two major sub-families the D_1_-like (D_1_R and D_5_R) and D_2_-like (D_2_R, D_3_R, and D_4_R) dopamine receptors, respectively [7].

As with all surface membrane receptors, the function of GPCRs is tightly linked to their intracellular trafficking. Their trafficking to the plasma membrane is needed for response to their extracellular ligand. Therefore, the appropriate delivery of GPCRs to the plasma membrane permits receptor/ligand interaction. Their subsequent internalization and re-insertion to the plasma membrane are of fundamental importance in the regulation of GPCR activity.

Several studies have shown that the C-terminus of D_1_R plays an important role in its plasma membrane trafficking. Vargas and von Zastrow [8] identified a novel endocytic recycling signal (amino acids 360–382) in the C-terminus of D_1_R. Bermak et al. [9] reported that a carboxy-terminal hydrophobic motif, F_333_XXXF_337_XXXF_341_, which is highly conserved among GPCRs, functioned independently as an endoplasmic reticulum (ER)-export signal for the D_1_R. It was further demonstrated that F_337_(X)_6_L_344_L_345_ plays a role in ER export of several GPCRs, including α_1_B-AR, α_2_B-AR, AT_1_R, and β_2_-AR [10], [11]. Furthermore, di-leucine mutant 5-HT_1A_R gets stuck in ER, indicating that the C-terminal di-leucine motif is involved in the proper folding of 5-HT_1A_R [12]. However, in other integral membrane proteins, the di-leucine motif typically plays a critical role in internalization and lysosomal or plasma membrane targeting [13], [14].

To characterize further the structural determinants involved in the trafficking of D_1_R from the ER to the plasma membrane, we generated a series of C-terminal mutants of D_1_R and analyzed their trafficking and function following agonist stimulation. Our results indicated that di-L motif is critical for the plasma membrane targeting of D_1_R. However, the internalized D_1_R continues to be functional, if stimulated by a cell permeable agonist.

## Materials and Methods

### DNA Constructs

**Figure 1:**
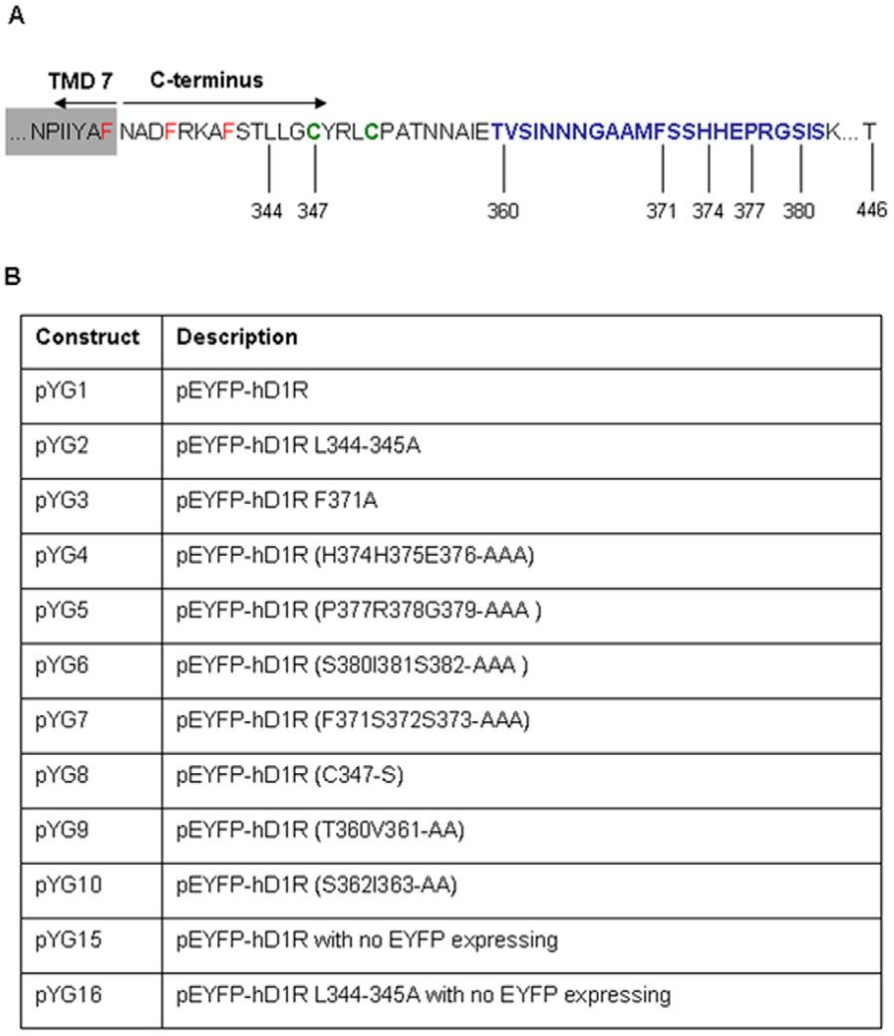
Cytoplasmic C-terminal domain of human D_1_R and constructs generated and used in this study. (A) The C-terminus of the human D_1_R contains a di-leucine motif (L344-345), two palmitoylation sites (in green, C347 and C351), and an endocytic recycling signal (in blue, amino acid residues 360–382). Three phenylalanine residues, in red, are highly conserved among GPCRs, function independently as an endoplasmic reticulum (ER)-export signal for the D_1_R. TMD7 stands for transmembrane domain 7. (B) 12 D_1_R constructs were generated using site-directed mutagenesis.

The complete coding sequence of human D_1_R was amplified by PCR with *Hind III* digestion site at the N-terminus and *Sac II* digestion site at the C-terminus, and then sub-cloned into the mammalian expression vector pEYFP-N1 (Clontech, Mountain View, CA) to generate pYG1 (pEYFP-hD_1_R). Then pYG1 was utilized in producing C-terminal mutants of D_1_R (pYG2-pYG16) (Fig. 1A and B), using the QuikChange site-directed mutagenesis kit (Stratagene, La Jolla, CA). To ensure that the YFP tagging does not interfere with the ligand binding, trafficking, or signaling of D_1_R, the constructs of wild-type D_1_R and di-L mutant that do not have YFP tags were also generated by putting a stop codon right before YFP in pYG1 and pYG2, respectively.

All constructs generated and used in this study (Fig. 1B) were confirmed by sequencing the complete open reading frame. All primer sequences are listed in Table 1.

**Table 1:** Primers for site-directed mutagenesis.

Primers	Sequences	Constructs generated
nYG7	5′-cggaaggcattttcaacggcggcaggatgctacagactttgccc-3′	pYG2
nYG9	5′-tggggcggcgatggcatccagccatcatg-3′	pYG3
nYG13	5′-gcgatgttttccagcgctgctgcgccacgaggctccatc-3′	pYG4
nYG14	5′-ccagccatcatgaggcagcagcctccatctccaaggag-3′	pYG5
nYG15	5′-catgagccacgaggcgccgccgccaaggagtgcaatctg-3′	pYG6
nYG12	5′-ggggccgcgatggctgccgcccatcatgagccac-3′	pYG7
nYG17	5′-caaccctcttaggaggctacagactttgc-3′	pYG8
nYG9	5′-gaataatgccatagaggcggcggctgccaataacaatggggcc-3′	pYG9
nYG10	5′-gccatagagacggtggctgccaataacaatggggc-3′	pYG10
nYG5	5′-cacccaaccccgcggtaggatccaccggtcgcc-3′	pYG15 (pYG1 as template)pYG16 (pYG2 as template)

### Cell Cultures and Transfections

Human embryonic kidney (HEK) 293 cells (ATCC, Manassas, VA) were maintained in Dulbecco's modified Eagle's medium supplemented with 10% heat-inactivated fetal bovine serum (ATCC, Manassas, VA) at 37°C with 95% air and 5% CO_2_. For transient transfections, cells were grown on the coverslips in 6-well plates and transfected at a ratio of 1 µg of plasmid DNA to 5 µL Lipofectamine 2000 (Invitrogen, CA) in 200 µL of serum-free medium, according to the manufacturer's protocol. The cells were processed ∼36 h after transfection for confocal microscopy or western blotting. Transfection efficiency was estimated to be same for each transfected plasmid based on the YFP fluorescence, using an epifluorescence microscope.

### Indirect Immunofluorescence

After transfection for ∼36 hours, HEK 293 cells grown on coverslips were rinsed in cold PBS and fixed with 4% paraformaldehyde for 20 min on ice, and then permeabilized with methanol for 20 min on ice. The following primary antibodies were used: monoclonal mouse anti-GM130 and monoclonal mouse anti-calnexin (BD Transduction Laboratories, Bellevue, WA), and polyclonal rabbit anti-EEA1 (Abcam, Cambridge, MA). Alexa Fluor 568 donkey anti-mouse IgG and Alexa Fluor 568 donkey anti-rabbit IgG were purchased from Invitrogen. Mounting medium with DAPI (Vector Laboratory, Burlingame, CA) was used. Images were obtained on Olympus Fluoview FV300 laser scanning confocal microscope equipped with a 40X 1.4 N.A. objective. FITC filter was used for imaging YFP-tagged proteins and the green color was selected as the pseudo color for YFP-tagged proteins in the figures. Images were processed using Adobe Photoshop. At least four studies were performed for any particular transfection. About 300 randomly selected transfected cells per coverslip were observed. The colocalization analysis was performed using Image J software.

### Cell Surface Biotinylation and Isolation

HEK 293 cells were cultured in 10-cm dishes and transfected with YFP-wild-type (wt) D_1_R or YFP-di-L D_1_R plasmid for cell surface biotinylation and isolation studies, using a kit from Thermo Scientific Pierce (Rockford, IL). In this study, one 10-cm dish of cells for each plasmid was used. Twenty-four hours after transfection the media were aspirated and cells quickly washed twice with 5 mL of ice-cold PBS per dish. Then, 5 mL of the biotin solution were added to each dish and gently agitated for 30 min at 4°C. At the end of 30 min, the reaction was stopped by the addition of 500 µL of quenching solution. The rest of the steps followed the manufacturer's protocol. The samples were subjected to SDS-PAGE, and then immunoblotted with anti-GFP antibody.

### Western Blotting

Cells grown in 6-well plates were washed twice with ice-cold PBS and then lysed in cold RIPA buffer containing 1 mM DTT and protease and phosphatase inhibitors (Sigma, St. Louis, MO) on ice for 10 min. The cells in each well were scraped and transferred to a 2 ml centrifuge tube. The lysates were centrifuged at 12,000 rpm in a pre-cooled centrifuge for 15 min and the supernatants were transferred to new tubes. The lysates were subjected to SDS-PAGE (4–12% gradient gel from Invitrogen) and immunoblotted with specific antibodies. Li-Cor Odyssey infrared imaging system (Li-Cor Biosciences, Lincoln, NE) was used for western blotting detection.

Goat polyclonal anti-GFP antibody purchased from Abcam (Cambridge, MA), which is designed to recognize all variants of GFP, was used for blotting all YFP-tagged proteins in this study. Rabbit anti-D_1_R antibody was from Origene (Rockville, MD). Secondary antibodies were all purchased from Li-Cor Biosciences (Lincoln, NE).

### State of Glycosylation Analysis

HEK 293 cells grown in the 6-well plates were transfected with same amount of pYG1 (YFP-wt D_1_R), pYG2 (YFP-di-L D_1_R), pYG15 (wt D_1_R), and pYG16 (di-L D_1_R). After an 8 hr transfection, the cells were treated with or without tunicamycin (TUN; 1 µg/ml; Sigma, St. Louis, MO) for another 16 hr. Then the cells were collected for western blotting following the steps mentioned above. To determine the oligosaccharides in the glycosylated D_1_R, 36 hr post-transfection, the cells were collected and denatured in glycoprotein denaturing buffers (New England Biolabs, Ipswich, MA) at 100°C for 10 min. Denatured sampled were digested with 500 U endoglycosidase H or peptide-N4-(N-acetyl-beta-glucosaminyl) asparagine amidase F (New England Biolabs) by incubation at 37°C for 1 hr. After digestion, samples were subjected to western blotting as mentioned above to analyze their state of glycosylation.

### Measurement of Cyclic AMP (cAMP) Production

HEK 293 cells were cultured in 6-well plates and then transfected with different D_1_R plasmids; non-transfected cells were included as negative control. 24 hr later, cells were treated with vehicle or the cell-membrane permeable D_1_R agonist (in the absence of D_5_R) fenoldopam (1 µM, 15 min) (Sigma, St. Louis, MO), or the cell-membrane impermeable D_1_R agonist (in the absence of D_5_R), A-68930 hydrochloride (1 µM, 15 min) (Sigma, St. Louis, MO). The cells were concomitantly treated with a phosphodiesterase inhibitor, IBMX (1 mM 3-isobutyl-1-methylxanthine, Sigma, St. Louis, MO). After the 15 min incubation period, 300 µL of 0.1 M HCl were added and incubated for another 20 min at room temperature. The amount of cAMP in each well containing ≥ 1 mg protein/ml was measured by an immunoassay kit (BioVision, Mountain View, CA), according to the manufacturer's protocol. Each treatment was performed in triplicate.

Data were expressed as mean + standard error. Significant differences between two groups were determined by Student's *t* test. A *P* value < 0.05 was considered significant.

## Results

As shown in Fig. 1A, the C-terminus of D_1_R contains a di-leucine motif (L344/345) that is very close to the 7^th^ transmembrane domain (TMD 7), two palmitoylation sites (C347/351), and an endocytic recycling signal sequence (T360—S382) identified by Vargas and von Zastrow [8]. Bermak et al. [9] also reported that a carboxy-termimal hydrophobic motif, FxxxFxxxF (F shown in red in Fig. 1A), which is highly reserved among GPCRs, functions independently as an ER-export signal for D_1_R. To study further the role of the C-terminus of D_1_R in its cellular trafficking and function, the di-leucine motif (L344/L345) and a series of amino acids within the endocytic recycling signal were mutated to alanine residues using site-directed mutagenesis; one palmitoylation site cysteine (C347) was replaced by a serine residue using the same technique (Fig. 1B). All constructs, except pYG15 and pYG16, were tagged with an enhanced YFP epitope at their C-termini in order to facilitate the visual evaluation of transfection efficiency and intracellular trafficking. All primer sequences are listed in Table 1.

### Di-leucine motif in the C-terminus of D_1_R is required for its plasma membrane trafficking

**Figure 2:**
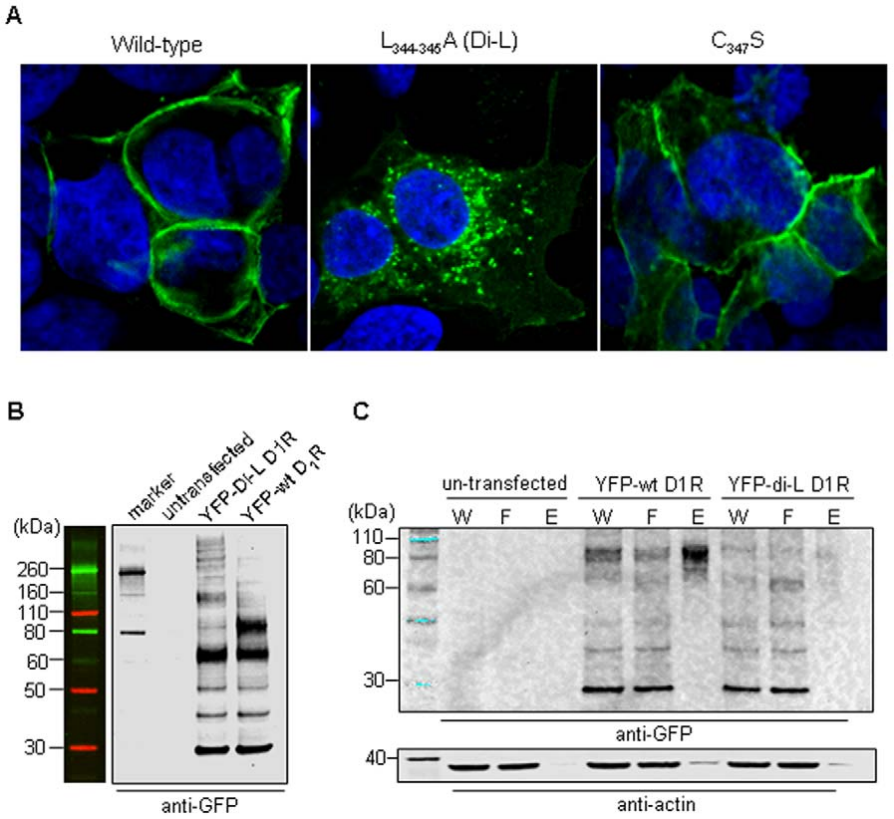
Trafficking of D1R C-terminal mutants. (A) HEK 293 cells transfected with YFP-tagged D_1_R plasmids grown on coverslips, and were fixed ∼36 hours post-transfection and subjected to confocal microscopy. Di-L and C347S were chosen as the representatives of the mutants. (B) HEK 293 cells were transfected with YFP-wt D_1_R and YFP-di-L D_1_R plasmids in 6-well plates. 36 hours after transfection, cells were washed twice with ice-cold PBS and then lysed in cold RIPA buffer containing 1 mM DTT and protease and phosphatase inhibitors on ice for 10 min. The cell lysates were subjected to SDS-polyacryalmide gel electrophoresis (4–12% gradient gel) and immunoblotted with goat-anti GFP antibody. (C) HEK 293 cells were cultured in 10-cm dishes and then transfected with same amount of YFP-wt D_1_R and YFP-di-L D_1_R plasmids. Cell surface biotinylation and isolation were performed using the commercial kit ∼24 h post-transfection. Non-transfected cells were included as a negative control. Samples were then immunoblotted with anti-GFP and anti-actin antibodies. W stands for whole cell lysate. F stands for flow through. E stands for elution.

We have studied the function of dopamine receptors in the kidney [3]–[5]. Therefore, HEK 293 cells were used as the *in vitro* model to express D_1_R. We have reported that HEK 293 cells do not express endogenously D_1_R or the other D_1_-like receptor, D_5_R [15]–[16], which was re-confirmed by immunoblotting and RT-PCR in the current report (data not shown). All constructs listed in Fig. 1B were transiently transfected into HEK 293 cells using Lipofectamine 2000 transfection reagent. The transfected cells were fixed ∼36 hrs post-transfection for confocal microscopy. As shown in Fig. 2A, the wild-type D_1_R was localized at the cell surface, which is consistent with previous reports, including those from our laboratory [15]. In contrast, di-leucine (Di-L) mutant D_1_R was localized in punctate intracellular compartments. All other mutants were trafficked to the plasma membrane as the wild-type D_1_R, including the C_347_S mutant in which a palmitoylation site was mutated, shown in Fig. 2A. Since the cell distribution of di-L mutant was very different from the rest of the mutants, we then detected its protein expression in transfected HEK 293 cells by western blot using anti-GFP antibody (Fig. 2B) and anti- D_1_R antibody (Supplemental Fig. S1). YFP-wt D_1_R showed a band of ∼90kDa and another band of ∼70kDa. By contrast, the di-L mutant also had a major band of ∼70kDa, but weak ∼90kDa. Both proteins had lager bands (≥160kDa), which could be the aggregation of the proteins. When blotted with anti-GFP antibody (Fig. 2B), both YFP-wt D_1_R and di-L mutant had a ∼30 kDa that may represent the soluble YFP (27kDa). The bands of ∼40–50 kDa were probably degraded D_1_Rs. We have previously reported [15] that the ≥90kDa bands are N-linked glycosylated proteins, located at the cell membrane. Glycosylation is necessary for the insertion of the D_1_R to the plasma membrane [17]. The C-terminal di-leucine mutant 5-HT_1A_ which is poorly glycosylated does not traffic to the plasma membrane [12]. Taken together, our studies suggest that di-L mutant D_1_R may not be fully glycosylated and fails to be targeted to the cell surface, suggesting that di-leucine motif in the C-terminus of D_1_R plays an important role in its plasma membrane trafficking.

To investigate further whether or not di-L mutant D_1_R could traffic to the plasma membrane, we studied the cell trafficking of cell surface-biotinylated pYG1 (pEYFP-wt D_1_R) or pYG2 (pEYFP-di-L D_1_R). Briefly, HEK 293 cells were transfected with YFP-wild-type (wt) D_1_R or YFP-di-L D_1_R plasmid for cell surface biotinylation and isolation studies using a kit from Thermo Scientific Pierce. Twenty-four hours after transfection, the media were aspirated and cells quickly washed twice with 5 mL of ice-cold PBS per 10-cm dish. Then, 5 mL of the biotin solution were added to each dish and gently agitated for 30 min at 4°C. The reaction was stopped by the addition of 500 µL of quenching solution. The rest of the steps followed the manufacturer's protocol. The samples were subjected to SDS-PAGE, and then immunoblotted with anti-GFP antibody.

As shown in Fig. 2C, actin protein, which was used as a loading control of the samples, indicated that the samples had same amount of whole cell lysate (lanes W) before the elution. Much less actin protein was detected in the eluted plasma membrane (lanes E) because the actin protein is predominantly expressed inside the cell. For the transfected cells, the soluble YFP bands (∼30 kDa) indicated similar transfection efficiency among the various samples. It is clear that much more YFP-wt D_1_R (lane E; ∼90 kDa) than YFP-di-L D_1_R proteins (lane E; ∼70 kDa) were eluted down after cell surface biotinylation and isolation. In other words, YFP-wt D_1_R protein was mostly expressed at the cell surface, but not the YFP-di-L D_1_R protein. This result is consistent with the fluorescence microscopy studies shown in Fig. 2A.

Therefore, these results indicated that di-L motif in the C-terminal of D_1_R plays a critical role in the plasma membrane targeting of D_1_R. The cell surface isolation results also suggested that the protein trafficked to the plasma membrane is glycosylated, consistent with our previous reports [15].

### Di-L mutant D_1_R is localized in early endosome

**Figure 3:**
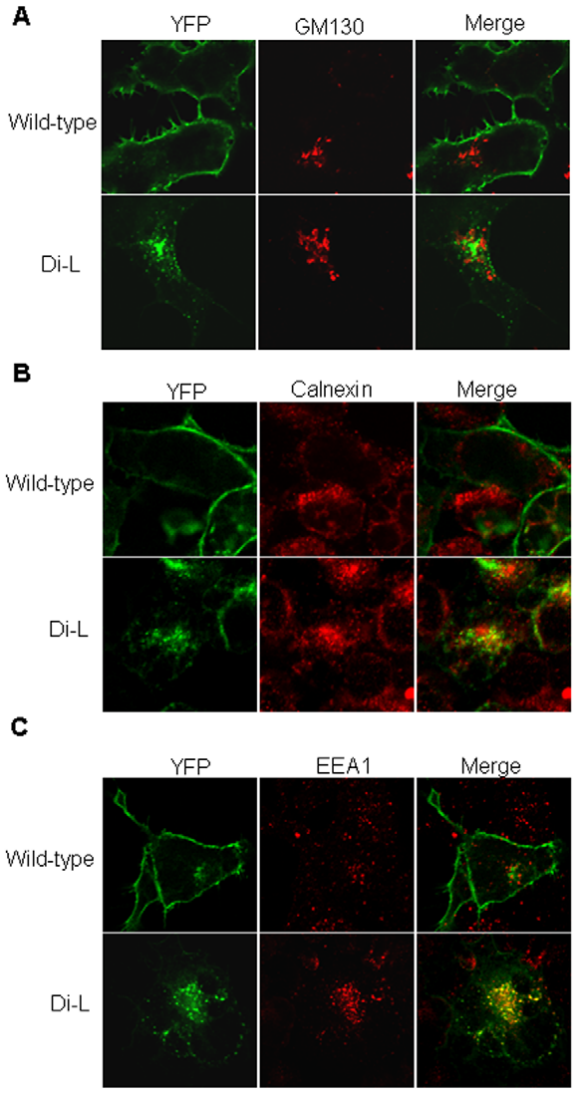
Di-L mutant D_1_R was co-localized with the early endosomes. (A) HEK 293 cells grown on the coverslips were transfected with YFP-wt D_1_R and YFP-di-L D_1_R. ∼36 h later, cells were rinsed in cold PBS and fixed by 4% paraformaldehyde for 20 min on ice, and then permeabilized by using methanol for 20 min on ice. The following primary antibodies were used: monoclonal mouse anti-GM130 (A), monoclonal mouse anti-calnexin (B), and polyclonal rabbit anti-EEA (C). Alexa Fluor 568 donkey anti-mouse IgG and Alexa Fluor 568 donkey anti-rabbit IgG were the secondary antibodies. Mounting medium with DAPI was used. Images were obtained on Olympus Fluoview FV300 laser scanning confocal microscope equipped with a 40X 1.4 N.A. objective. Images were processed using Adobe Photoshop. For each transfection experiment, at least four times were performed. For each coverslip, 200∼300 transfected cells were observed.

Since di-L mutant D_1_R was mainly localized in the intracellular compartments, we determined the identity of these compartments. We immunostained pYG2 (di-L mutant D_1_R)-transfected HEK 293 cells with the *cis*-Golgi marker, GM130, the endosomal marker, EEA1, and the ER marker, calnexin. Di-L mutant D_1_R did not colocalize with GM130 (Fig. 3A), colocalized partially with calnexin (Fig. 3B), but colocalized mostly with EEA1 (Fig. 3C). By using the Image J software, we calculated that ∼20% of YFP-di-L D_1_R proteins were colocalized with calnexin; but ∼80% of YFP-di-L D_1_R proteins were colocalized with EEA1, indicating that most of YFP-di-L D_1_R proteins were localized in the early endosomes. Each transfection was repeated four times, and 200∼300 cells were observed in each coverslip. These data indicated that di-L mutant could be transported from ER to the Golgi region and subsequently to the endosomal compartments. However, the di-L mutant D_1_R failed to be inserted into the plasma membrane which is the final destination of wt D_1_R, suggesting that the di-L motif in the C-terminus of D_1_R plays a critical role in the plasma membrane targeting of D_1_R.

### Glycosylation state of D_1_R

Given that the activity of several GPCRs depends on their glycosylation status and plasma membrane trafficking [18], we investigated the glycosylation state of both wt D_1_R and di-L mutant D_1_R in transfected cells.

**Figure 4:**
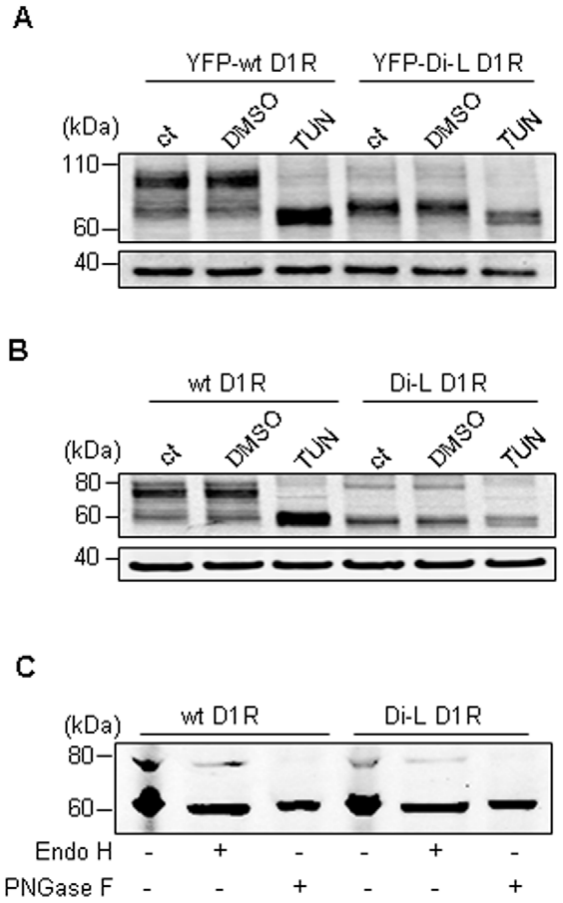
Glycosylation states of wt D_1_R and di-L mutant D_1_R were analyzed by glycosylation inhibitors. (A) After an 8 hr transfection, the HEK 293 cells heterologously expressing the YFP-wt D_1_R and YFP-di-L D_1_R proteins were treated with or without tunicamycin (1 µg/ml) for another 16 hr. Then the cells were collected for western blotting. (B) After an 8 hr transfection, the HEK 293 cells heterologously expressing the wt D_1_R and di-L D_1_R proteins were treated with or without tunicamycin (1 µg/ml) for another 16 hr. Then the cells were collected for western blotting to compare with Fig. 4A. (C) To determine the oligosaccharides in the glycosylated D_1_R, 36 hr post-transfection, the cells heterologously expressing wt D_1_R and di-L D_1_R proteins were collected and denatured in glycoprotein denaturing buffers at 100°C for 10 min. Denatured sampled were digested with 500 U endoglycosidase H or peptide-N4-(N-acetyl-beta-glucosaminyl)asparagine amidase A by incubation at 37°C for 1 hour. After digestion, samples were subjected to western blotting to analyze their state of glycosylation.

HEK 293 cells heterologously expressing the YFP-wt D_1_R and YFP-di-L D_1_R proteins were treated with the N-lined glycosylation inhibitor tunicamycin (TUN). YFP-wt D_1_R migrated as a band of ∼90 kDa and a band of ∼70 kDa in untreated cells (Fig. 4A, ct) and in vehicle (DMSO)-treated cells, similar to those shown in Fig. 2B. After tunicamycin treatment, YFP-wt D_1_R migrated mainly as a band of ∼70 kDa; the band of ∼90 kDa was much less compared to that in the non-TUN-treated cells, indicating that the ∼90 kDa YFP-wt D_1_R was glycosylated and the protein of ∼70 kDa was unglycosylated. In the untreated and vehicle (DMSO)-treated YFP-di-L D_1_R cells (Fig. 4A), the major band was ∼70 kDa with a minor band of ∼90 kDa, which were also similar to those shown in Figure 2B. Tunicamycin treatment minimally affected the ∼90 kDa YFP-di-L, suggesting that a small portion of di-L mutant D_1_R was also N-linked glycosylated. The YFP tag did not interfere with the post-translational modification of D_1_R proteins because similar results were obtained using non-tagged D_1_R and di-L D_1_R (Fig. 4B); the bands were lower than those in Fig. 4A because of the absence of the YFP tag.

It has been known that the majority of mature glycoproteins traversing the Golgi are N-glycosylated, whereas those residing in the ER are high-mannose glycosylated [19]. Therefore, we investigated the type of glycosylation of D_1_R by treating the denatured cell lysates with Endo H to remove high-mannose N-glycosylation, or peptide N-glycosidase F (PNGase F) to remove both high-mannose and complex N-glycosylation (Fig. 4C). After Endo H treatment, the ∼80 kDa band (fully glycosylated non-tagged D_1_R) was still visible in both wt and di-L mutant D_1_R, suggesting that the D_1_R was not totally high-mannose N-glycosylated. By contrast, PNGase F treatment resulted in the migration of both wt D_1_R and di-L mutant D_1_R at the ∼60 kDa, which corresponded to unglycosylated non-tagged D_1_R protein, indicating that both wt and partial di-L mutant D_1_R goes through complex glycosylation, which occurs only in the Golgi apparatus [20].

### Cyclic AMP assay

To test the functionality of di-L mutant D_1_R and other C-terminal mutants, we measured the accumulation of cyclic AMP (cAMP) in the transfected cells that were treated with vehicle (water) or fenoldopam, D_1_R agonist. To achieve similar transfection efficiency, HEK 293 cells were transfected with same amount of each plasmid listed in Fig. 1B. We also visualized the cells under the fluorescence microscope to further confirm their transfection efficiencies (data not shown) before the treatment.

**Figure 5:**
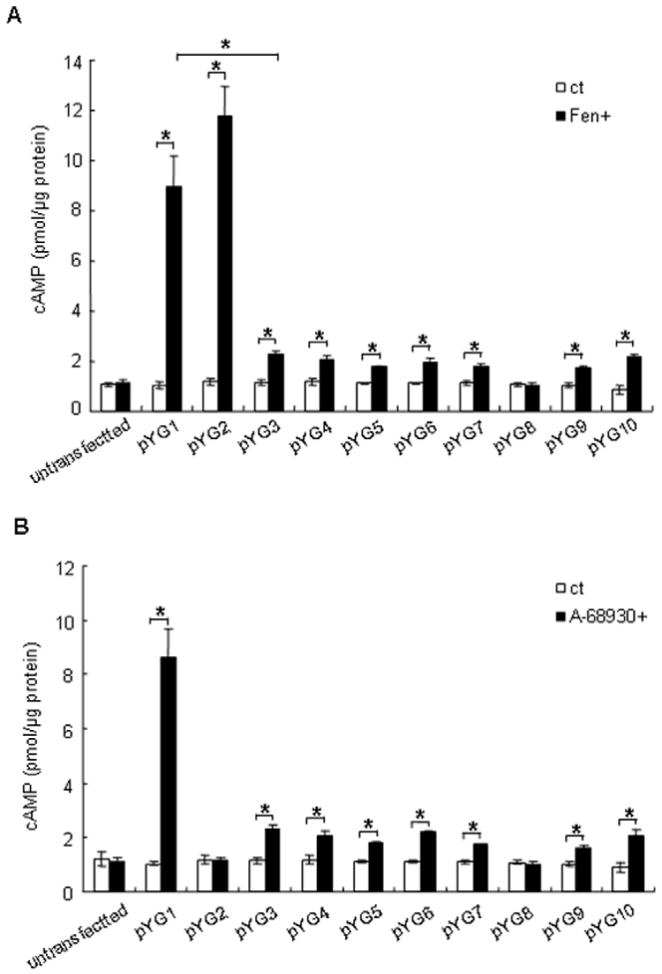
Di-L mutant D_1_R has an impaired ability to increase cAMP accumulation activity in response to A-68930. HEK 293 cells were transfected with same amount of each plasmid as indicated. 24 hours later, cells were treated with or without 1 µM of fenoldopam (A) or 1 µM of A-68930 for 15 min. The cAMP direct immunoassay kit was used for measuring the production of cAMP. Each treatment was performed in triplicate. Data were expressed as mean ± standard error. Significance between groups was determined by Student's *t* test. A *P* value < 0.05 was considered significant.

As stated above, RT-PCR and immunoblotting studies confirmed our previous reports that HEK 293 cells used in this study do not endogenously express either D_1_R or D_5_R (data not shown). Therefore, as we expected, fenoldopam (1 µM, 15 min) did not affect the cAMP accumulation in untransfected HEK 293 cells (Fig. 5, untransfected), however, the wt D_1_R (pYG1)-transfected HEK 293 cells had a marked increase in cAMP accumulation with fenoldopam stimulation (Fig. 5A). Surprisingly, the accumulation of cAMP in di-L mutant D_1_R (pYG2)-transfected cells in response to fenoldopam was similar to wt D_1_R, although this mutant failed to traffic to the cell surface (Figs. 2 and 3). Since fenoldopam is a relatively cell-membrane permeable agonist (soluble to 12.2 mM in water, but >31.1mM in DMSO, according to the datasheet from the manufacturer), we hypothesized that fenoldopam that entered the cell freely could bind to di-L D_1_Rs inside the cell but remain functional. To test our hypothesis, we tested the ability of relatively cell-membrane impermeable D_1_R agonist, A-68930 (soluble to 50 mM in water, according to the datasheet from the manufacturer) to increase cAMP accumulation in these cells (Fig. 5B). In this experiment, cAMP accumulation did not increase in di-L mutant D_1_R expressing cells. These results confirmed our hypothesis and suggested that di-L motif plays an important role in the plasma membrane targeting of D_1_R and its response to extracellular agonist stimulation.

When palmitoylation site (Cys347) was mutated to serine residue (347 C>S), accumulation of cAMP was not increased by fenoldopam, indicating that palmitoylation of D_1_R is necessary for agonist action (Fig. 5A, pYG8). The other mutant D_1_Rs (pYG3, 4, 5, 6, 7, 9, 10) in which the mutated sites were within the endocytic recycling signal had an increase in cAMP accumulation in response to fenoldopam stimulation, albeit to a much lower extent than those observed in wt D_1_R. These results could be taken to suggest that these mutants could be internalized as the wt after the agonist stimulation, but are minimally recycled back to the plasma membrane and therefore, respond minimally to continuous agonist stimulation, thus the increase in cAMP was much lower than that of the wt. However, this is unlikely because di-L mutant D_1_R which is minimally trafficked to the plasma membrane had a marked increase in cAMP response to the cell membrane permeable but not cell membrane impermeable agonist. Rather, these mutations interfered with the intrinsic ability of D_1_R to respond to agonist stimulation, the mechanisms of which remain to be determined.

## Discussion

In general [21], GPCRs initially reside in the ER after synthesis, where they undergo processing and folding guided by chaperone and quality-control proteins. Following their exit from the ER, GPCRs transit through the Golgi apparatus for additional modifications. On the outer edge of the Golgi, GPCRs are packaged in exocytic transport vesicles and enter the endosomal system, where they are subsequently targeted to the plasma membrane. After the extracellular agonist stimulation, the activated GPCR acts as a guanine nucleotide exchange factor, catalysing the exchange of GDP for GTP on the Gα subunit and inducing dissociation of the Gα and Gβγ subunits from each other and from the GPCR. Activated GTP α subunits of which there are multiple subtypes, including Gαs, Gαi, Gα12/13 and Gαq, subsequently bind to and regulate the activity of effectors such as adenylyl cyclase. Agonist binding also promotes GRK-mediated phosphorylation of the cytoplasmic surface of GPCR and subsequent β-arrestin translocation and binding to the receptor. β-Arrestin binding, in turn, facilitates the subsequent recruitment of AP-2 and clathrin and GPCR inclusion in clathrin-coated pits before endocytosis via clathrin-coated vesicles. Most internalized receptors may be either recycled to the plasma membrane or sorted to lysosomes and proteasomes for degradation. The early endosomes involved in GPCRs trafficking to the plasma membrane are morphologically and functionally distinct and can be identified by association with small GTPases called Rabs [22]. Furman et al. (2009) also showed for the first a functional role of Rab 11 in the trafficking of dopamine receptor to the plasma membrane [23], whereas sorting nexin 1 (SNX1) has more recently been shown to play a role in endosomal to lysosomal GPCR sorting [24]. As a GPCR, it is not clear yet how D_1_R traffics in the cell and how its trafficking relates to the function, although it has been reported that the C-terminus of D_1_R is very important for its trafficking and function [8]–[9].

In this study, we investigated the plasma membrane trafficking and function of a series of C-terminal mutant D_1_Rs in transfected HEK 293 cells. Our results interestingly showed that when the di-leucine motif (L344-345) at the C-terminus of D_1_R was mutated, the mutant protein was not able to traffic to the plasma membrane; instead, it was localized in the early endosome. This data suggested that this C-terminal di-L motif is a plasma membrane targeting signal. Kim et al. [25] reported that the cell surface expression of a deletion mutant D_1_R (truncated at position 347) was diminished relative to the wild-type. In their mutant, di-L motif was intact, but the mutant could not get to the cell surface. Combined with our results, we propose that the di-L motif (L344-345) is required for the cell surface targeting of D_1_R, but is not the only signal for this trafficking. This C-terminal di-L motif is highly conserved in GPCRs, but replacement of di-leucine motif in the C-terminus of the β_2_-AR by alanines resulted in a marked reduction in internalization [26], suggesting that di-leucine motif plays a critical role in the endocytosis of β_2_-AR, which is similar to its function in other membrane proteins [27]–[28].

Another common structural theme among GPCRs is palmitoylation of one or more sites of the C-terminal tail near the seventh transmembrane domain [29]. It has been shown that D_1_R has two palmitoylation sites at positions 347 and 351 in the carboxyl tail [30]–[31]. The substitution of Cys347 with a serine led to a diminished ability to activate adenyly cyclase, indicating that Cys347 is important for D_1_R in maintaining the conformation for antagonist binding and is essential for D_1_R's agonist-induced desensitization, however, the pharmacological and functional properties of C351S mutant were similar to that of wild-type D_1_R [30]. In our study, we mutated Cys347 to serine residue (construct of pYG8); the mutant protein was localized at the cell surface (Fig. 2A), but failed to increase the accumulation of cAMP in response to fenoldopam or A-68930 stimulation (Fig. 5). These results further suggested that Cys347 is important for D_1_R trafficking and responsiveness [32].

It has been known that N-glycans attached to the membrane proteins can act as a plasma membrane sorting signal, but it does not ensure their distribution to the plasma membrane. For example, inhibiting the glycosylation of D_1_R deceased the cell surface trafficking of D_1_R [17], however, these results contrast with those of Karpa *et al.* [33], who found that N-linked glycosylation was not required for D_1_R localization to the plasma membrane. The reason(s) for this discrepancy is not clear. In this study, we found that the glycosylation of YFP-di-L D_1_R was markedly decreased and markedly limited expression at the cell surface compared with the wild-type (Figs. 2 & 4), which is consistent with the finding of Free et al.[17], indicating that the glycosylation is required for the cell surface trafficking of D_1_R. To determine the type of N-linked glycosylation of D_1_R, we treated the cells with Endo H and PNGase F; PNGase F can remove all N- linked carbohydrates (complex N-glycans) without regard to type, whereas Endo H removes only high mannose and some hybrid types of N-linked carbohydrates. The results in Fig. 4C clearly showed that the glycosylation of D_1_R is that of complex N-glycosylation. This glycosylation state may explain why YFP-di-L D_1_Rs (Fig. 3), which are minimally glycosylated were localized in the early endosome but not in the Golgi or endoplasmic reticulum (ER), because the majority of mature glycoproteins that traverse the Golgi carry complex N-glycans [19].

The function of each mutant D_1_R protein was studied by their ability to increase cAMP accumulation after agonist stimulation. Surprisingly, di-L mutant D_1_R (pYG2 in Fig. 5A), which was not localized at the cell surface membrane but rather inside the cell, increased cAMP accumulation in response to fenoldopam to a similar extent as wt D_1_R. Because fenoldopam (selective D_1_R agonist, in the absence of D_5_R) is relatively cell-membrane permeable we hypothesized that fenoldopam could bind to the di-L D_1_Rs that continue to be functional inside the cell. However, when the cells were treated with A-68930 (also a selective D_1_R agonist, in the absence of D_5_R), which is a relatively cell-membrane impermeable, the accumulation of cAMP was not affected. These data indicated that the response of di-L mutant D_1_R to extracellular agonist stimulation was impaired because of a failure of di-L mutant D_1_R to be trafficked to the plasma membrane. The mutants other than pYG2 had a limited cAMP response to both fenoldopam and A-68930, probably because of impairment of function, not necessarily related to cell surface membrane trafficking. It could be that they have greatly diminished resensitization or they cannot find Gs and/or cylase in early/recycling endosomes.

In summary, di-L motif (L344-345) at the C-terminus of D_1_R is required for its plasma membrane trafficking and glycosylation. Further investigations may reveal how di-L motif is involved in the sorting of D_1_R.

## Supporting Information

Figure S1 **Western blots using anti- D1R antibody.** HEK 293 cells were transfected with YFP-wt D_1_R and YFP-di-L D_1_R plasmids in 6-well plates. 36 hours after transfection, cells were washed twice with ice-cold PBS and then lysed in cold RIPA buffer containing 1 mM DTT and protease and phosphatase inhibitors on ice for 10 min. The cell lysates were subjected to SDS-polyacryalmide gel electrophoresis (4–12% gradient gel) and immunoblotted with rabbit anti-D_1_R antibody.
(TIF)
