# Supplementary figures and images for: C-Terminal Di-leucine Motif of Dopamine D1 Receptor Plays an Important Role in Its Plasma Membrane Trafficking
Source: PLoS One. 2011 Dec 19;6(12):e29204. doi: 10.1371/journal.pone.0029204 (PMC3242775; doi:10.1371/journal.pone.0029204)

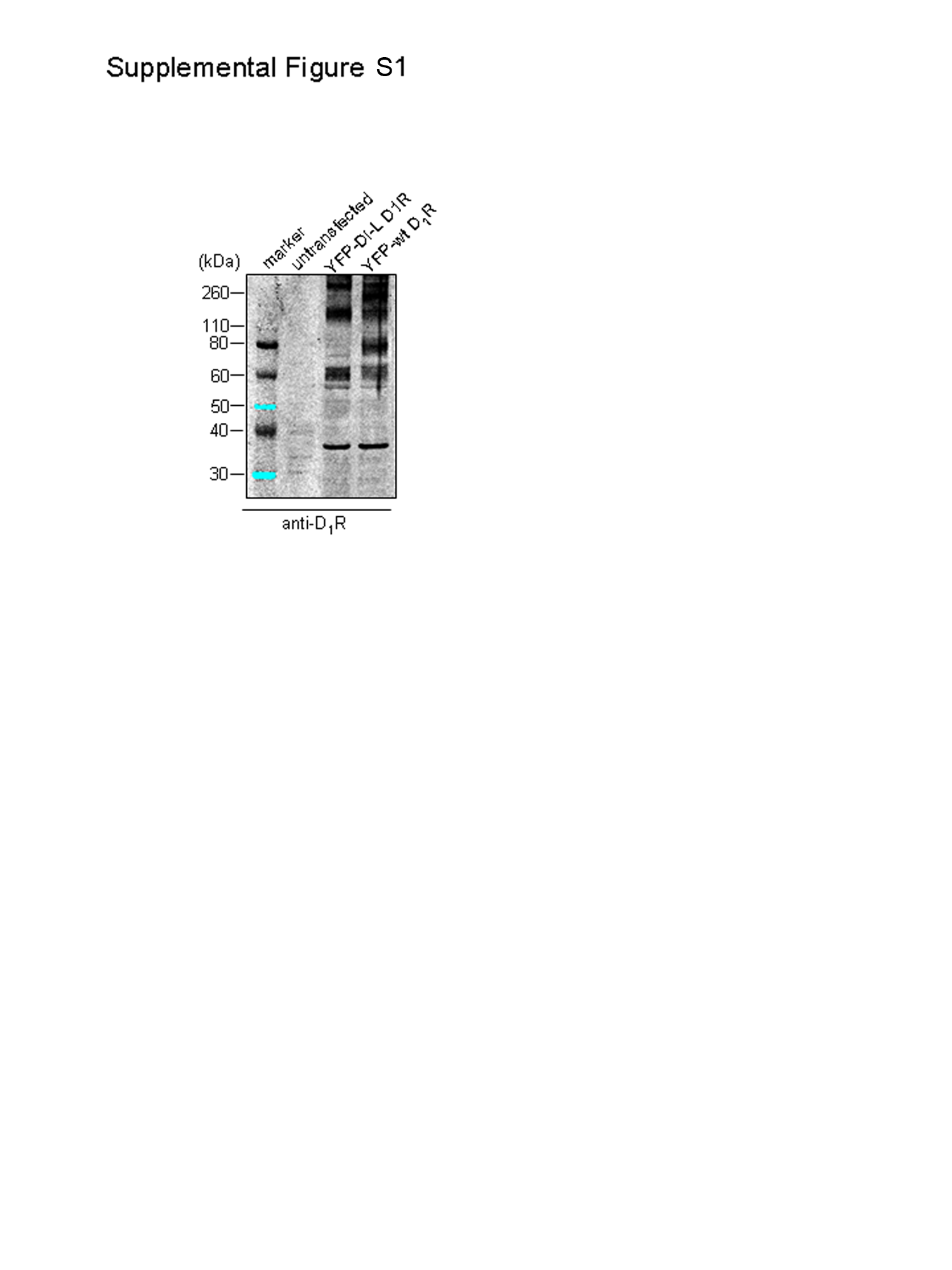

Supplement: Figure S1 — Western blots using anti- D1R antibody. HEK 293 cells were transfected with YFP-wt D1R and YFP-di-L D1R plasmids in 6-well plates. 36 hours after transfection, cells were washed twice with ice-cold PBS and then lysed in cold RIPA buffer containing 1 mM DTT and protease and phosphatase inhibitors on ice for 10 min. The cell lysates were subjected to SDS-polyacryalmide gel electrophoresis (4–12% gradient gel) and immunoblotted with rabbit anti-D1R antibody. (TIF) [file pone.0029204.s001.tif]
